# 954. The Impact of Targeted Education on Burn Unit Nurse Resident Confidence and Turnover

**DOI:** 10.1093/jbcr/irag033.521

**Published:** 2026-04-06

**Authors:** Nicole Ragon, Eric Jensen

**Affiliations:** CHI Health St Elizabeth Burn Center, Lincoln, NE; CHI Health St Elizabeth Burn Center, Lincoln, NE

## Abstract

**Introduction:**

An acute care hospital that has experienced an 18.2% first-year turnover rate for burn nurses, exceeding the national average of 16.4%. This high turnover costs hospitals significantly. In 2022, the hospital started a National Nurse Residency Program (NNRP) to boost readiness, retention, and job satisfaction, which has been shown to improve nurse confidence and competence. This project aims to improve new Burn nurse resident confidence and reduce first-year turnover by integrating specialized burn-specific curricula into the residency program, measured by the Casey Fink (CF) survey.

**Methods:**

This quality improvement initiative will assess the impact of specialized burn education on new nurse residents. The CF Survey, measuring clinical preparedness, will be administered at 30 days, 6 months, and 12 months throughout NNRP. Burn-specific education, guided by American Burn Association competencies, will be integrated into the Critical Care NNRP curriculum. Data analysis will compare Role Confidence, Role Satisfaction (from CF surveys), and 12-month turnover rates of the current cohort against previous Burn NNRP cohorts and historical data.

**Results:**

Previous cohorts showed average Role Confidence scores of 2.84 and Role Satisfaction scores of 3.25 in the initial survey. After six months, these averages shifted to 3.18 for Role Confidence and 2.40 for Role Satisfaction. At the one-year mark, Role Confidence averaged 3.09, and Role Satisfaction was 2.56. The Burn unit's first-year turnover rate is 18.2%, which exceeds the national average. The current cohort's initial survey revealed a 4.43% increase in Role Confidence and a 7.62% increase in Role Satisfaction. Subsequent CF Surveys will be benchmarked against previous cohort outcomes, with the aim of achieving a statistically significant improvement and a reduction in nurse turnover.

**Conclusions:**

The initial findings from the current cohort's Casey Fink (CF) survey are encouraging. While these early improvements are promising, the long-term impact on both confidence and satisfaction, as well as the outcome of first-year turnover reduction, remains under evaluation. Subsequent CF surveys at the 6-month and 12-month marks will be essential for benchmarking against historical data and previous cohort outcomes. The ultimate success of this initiative will be determined by achieving statistically significant improvements in these metrics and a measurable reduction in the burn unit's first-year nurse turnover rate.

**Applicability of Research to Practice:**

This project applies evidence-based education to improve burn nurse retention, confidence, and satisfaction. Using American Burn Association competencies, it develops targeted education and rigorously evaluates its effectiveness with the CF Survey and 12-month turnover rates. Integrating this into the existing NNRP ensures sustainability and translates best practices into better clinical preparedness and retention.

**Funding for the study:**

N/A.

